# Adolescent Alcohol Exposure Produces Protracted Cognitive-Behavioral Impairments in Adult Male and Female Rats

**DOI:** 10.3390/brainsci10110785

**Published:** 2020-10-28

**Authors:** Victoria Macht, Natalie Elchert, Fulton Crews

**Affiliations:** 1 Bowles Center for Alcohol Studies, University of North Carolina, Chapel Hill, NC 27599, USA; vmacht@email.unc.edu (V.M.); nelchert@live.unc.edu (N.E.); 2 Department of Pharmacology, University of North Carolina, Chapel Hill, NC 27599, USA; 3 Department of Psychiatry, University of North Carolina, Chapel Hill, NC 27599, USA

**Keywords:** ethanol, working memory, sex differences, development, learning, memory, spatial navigation, dominance, perseveration, aggression

## Abstract

Binge drinking is common in adolescence. Rodent studies modeling adolescent binge drinking find persistent effects on the brain’s physiology, including increased expression of neuroimmune genes, impaired neurogenesis, and changes in behavioral flexibility. This study used females and males to investigate the effects of adolescent intermittent ethanol (AIE) on a battery of behaviors assessing spatial navigation using a radial arm water maze, working memory using the Hebb-Williams maze, non-spatial long-term memory using novel object recognition, and dominance using a tube dominance test. Results indicate that AIE impairs adult acquisition in spatial navigational learning with deficits predominantly driven by females. Surprisingly, AIE slowed the transition from random to serial search strategies in both sexes, suggesting AIE impairs flexibility in problem-solving processing. In the Hebb-Williams maze working memory task, adult AIE rats exhibited deficits in problem solving, resulting in more errors across the 12 maze configurations, independent of sex. Conversely, AIE decreased dominance behaviors in female rats, and at 7 months post-alcohol, female AIE rats continued to exhibit deficits in novel object recognition. These results suggest that cognitive-behavioral alterations after adolescent binge drinking persist well into middle age, despite abstinence. Future studies should focus on intervening treatment strategies in both females and males.

## 1. Introduction

The majority of all alcohol intake by adolescents is consumed in patterns of binge exposure (>5 drinks/session for men; >4 drinks/session for women) [1]. Binge drinking is associated with a host of acute negative behavioral outcomes, including increased risk taking, violence, teenage pregnancy, and other adverse mental health conditions [2,3,4]. However, because adolescence is a critical period of brain maturation, it is becoming increasingly apparent from preclinical studies that chronic complications of binge alcohol exposure after adolescence are important areas of investigation. For example, preclinical studies have highlighted that the cognitive and neurological consequences of adolescent binge drinking can persist into adulthood, suggesting that binge drinking during adolescence is changing the trajectory of brain development in a permanent way [5,6,7].

This pattern of alcohol intake is evident in both males and females, with almost 20% of male and female 12th graders having engaged in binge drinking in the last two weeks [8]. Previous preclinical studies have demonstrated that adolescent intermittent ethanol (AIE) exposure impacts performance in a variety of behavioral tasks, included inducing deficits in the Barnes and Morris water maze tasks [6,9,10], novel object recognition (NOR) [11], and social interaction tasks [12]. To expand on these results, the current study assessed the impact of AIE on learning and memory in male and female rats across a variety of cognitive domains, including spatial, non-spatial, and social domains, using both aversive and appetitive motivating factors, and working versus long-term memory components. Behavioral tests included the radial arm water maze (RAWM), Hebb-Williams maze, tube dominance test, and novel object recognition test, respectively. These tests were performed starting more than a month after the end of adolescent alcohol exposure and continuing across adulthood and into middle age with the aim to assess the longitudinal nature of cognitive-behavioral deficits after AIE.

The primary hypothesis of the current study was that AIE would impair behavioral performance in working and long-term memory tasks as well as dominance behaviors across adulthood in both male and female rats and that these effects would persist into middle age. In addition, as deficits in behavioral flexibility following AIE have been found in many preclinical studies as well as in humans with alcohol use disorder [1], and as some studies have demonstrated inefficient problem-solving strategies in male rats [13], we further hypothesized that AIE would produce deficits in the flexibility of search strategies employed in both sexes. We also hypothesized that AIE-treated female rats would be more sensitive to deficits in social dominance, as human clinical depression is almost twice as prevalent in women as in men [14] and increased submissive behaviors are associated with increased risk for developing depressive-like phenotypes [15,16,17,18,19]. Lastly, we hypothesized that both males and females would exhibit long-term memory deficits in NOR that persist until middle age, extending previous findings longitudinally and across both sexes [5,11,20].

In brief, results from the current study indicate that AIE exposure impairs cognitive flexibility across a variety of behavioral paradigms, including the RAWM and Hebb-Williams maze. However, current results also indicate that females are particularly sensitive to cognitive deficits after AIE, with impairments in NOR evident even in middle age. Females also appear more sensitive to shifts in social hierarchy after AIE, with AIE progressively increasing submissive phenotypes in the tube dominance test. Importantly, these persistent deficits after AIE are evident across a battery of behaviors and are long-lasting despite abstinence.

## 2. Materials and Methods

### 2.1. Animals

Male and female Long Evans rats were bred in-house at the University of North Carolina at Chapel Hill. Housing conditions for all rats were maintained at 20 °C in a humidity-controlled vivarium with a 12/12 light dark cycle with lights on at 7:00 a.m. Rats were granted *ad libitum* access to food and water unless otherwise specified. All experimental procedures were approved by the Institutional Animal Care and Use Committee at the University of North Carolina at Chapel Hill and conducted in accordance with the National Institutes of Health regulations on rodent research.

### 2.2. Adolescent Intermittent Ethanol (AIE) Exposure Paradigm

**Figure 1:**
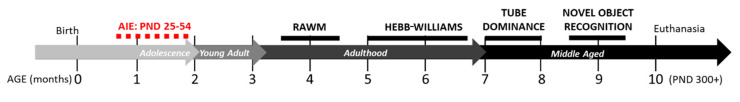
Experimental timeline of adolescent intermittent ethanol (AIE) and behavior tests across the lifespan. Long Evans rats underwent intermittent gavage with either water (CON) or 5 g/kg ethanol (AIE) across adolescence (postnatal day (PND) 25–54). After two months of rest, rats began a battery of behavioral tests that included spatial (radial arm water maze, Hebb-Williams maze) and non-spatial (novel object recognition) memory tasks as well as an assessment of dominance behaviors (tube dominance test). In every test, females and males were tested separately. Rats were allowed at least two weeks of recuperation between each test. There were 14 CON rats (*n* = 8 male, *n* = 6 female) and 13 AIE rats (*n* = 7 male, *n* = 6 female).

At postnatal day (PND) 21, rats were weaned and same-sex pair housed in a split litter design. On PND 25, rats were randomly assigned to either the condition of control (male CON, *n* = 8; female CON, *n* = 6) or adolescent intermittent alcohol exposure (male AIE, *n* = 7; female AIE, *n* = 6). From PND 25–54, rats were subjected to 2-day on/2-day off intermittent intragastric gavage of either water (CON) or 5 g/kg ethanol (AIE), as previously described [5]. Tail bleeds were performed on the last day of the AIE paradigm, one-hour post-gavage, to confirm blood ethanol content (BEC). Mean BEC for both males and females was 188.9 ± 16.7 mg/dL, consistent with previous studies [5,10,21]. Following the end of the adolescent exposure paradigm, rats rested in the vivarium for two months, until beginning a series of behavioral experiments (see Figure 1 for an experimental timeline). As several of the behavioral measures employed have stressful components, rats were allowed at least two weeks of rest between sets of behavioral tests to mitigate potential compounding stress effects. For all testing, males and females were run on separate days to avoid confounding sex-related olfactory cues.

### 2.3. Radial Arm Water Maze

The RAWM was selected because it is a powerful test of long-term memory in combination with allocentric spatial navigation; it takes advantage of the spatial complexity of the radial arm maze with the rapid learning induced by the aversive nature of the stimulus in the Morris water maze, and has been used successfully in a variety of fields to highlight subtle differences in spatially based learning and memory in aging [22], after predator odor stress [23], and with Alzheimer’s models [24]. In addition, the RAWM allows for assessments of non-spatially-based search strategies (i.e., random, serial) without the confounds of thigmotaxis, which are more difficult to assess in a traditional Morris water maze. The apparatus consisted of a 150 cm plastic pool, inside which an 8-arm gray radial arm Kydex insert was placed. The apparatus also included a white Kydex sheet on the bottom to enhance contrast and improve software tracking capability. The tub was filled with water (23 °C) to a depth of 35 cm. A transparent, circular Plexiglas escape platform was submerged in one arm of the maze, approximately 2 cm below the water surface. Extradimensional cues were placed around the room to facilitate spatial navigation. Rats were given five trials per day over the course of 4 days with 60-minute inter-trial intervals. Start locations were randomized with the criteria that (1) the start location was not juxtaposed to the arm with the escape platform, (2) no two trials in a given day started from the same location, and (3) each day consisted of a randomized pattern of start locations. Rats were gently placed into the water of the RAWM facing the wall. Each trial lasted no more than 120 s, after which the rat was gently guided to the platform in the event of a failed trial. Rats were towel-dried and returned to their home cage between trials. Males and females were tested on separate days, between which the water maze was emptied and cleaned to avoid confounding olfactory cues between sexes.

Behavior was tracked with the automated tracking software Ethovision, which provided latency to the escape platform and frequency of incorrect arm entries (i.e., errors). Errors were defined as either long-term memory errors (i.e., entering an arm without the escape platform) or working-memory errors (reentering an incorrect arm within a trial). To assess whether performance in the RAWM was linked with differences in non-spatial search strategies being used, entry pattern into arms was recorded by a blind observer and strategies were identified as either predominantly serial (sequential investigation of adjacent arms in a clockwise or counterclockwise manner) or random (unsystematic investigation of arms). Search strategy was assessed only on the first day of RAWM training as improvements in spatial memory across groups during subsequent days interfered with this analysis.

### 2.4. Hebb-Williams Maze

**Figure 2:**
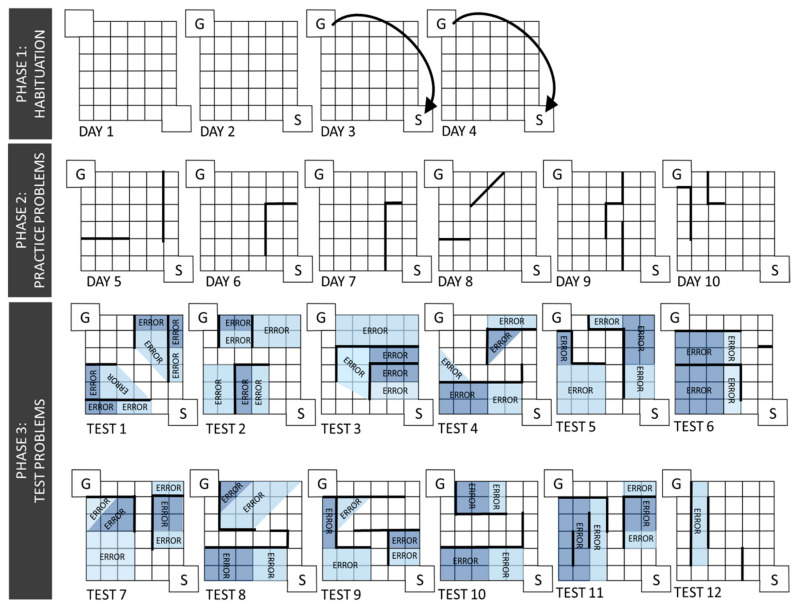
Hebb-Williams maze configurations. The above maze configuration and testing procedures were adapted from Rabinovitch and Rosvold [25]. Testing consisted of three phases: habituation, practice, and test. During the habituation phase, rats were acclimated to the testing apparatus and trained to run from the start box (S) to the goal box (G). Then, rats went through 6 different practice problems, followed by 8 runs each of 12 test problems. Each test problem has error zones, and an error was scored when the rat entered the incorrect zone, as determined by a blind observer. Each problem varied in difficulty level so that easy and difficult problems were dispersed throughout the testing paradigm.

The Hebb-Williams maze is a working memory task adapted from Rabinovitch and Rosvold (1951) that assesses problem-solving skills in rodents and has been standardized and likened to a rodent test of intelligence [25]. This maze is unique in that it is directly adaptable to clinical settings using virtual reality, making this one of the only behavioral tasks for which the exact same maze parameters can be used to assess cognitive function in rodents and humans [26]. The maze is a 60 cm × 60 cm open field apparatus with a start box and goal box on opposite diagonals. A removable Plexiglas lid is attached to the top of the maze to discourage rearing exploratory behavior. The white flooring is divided into a series of black-lined 12 cm squares. Movable vertical barriers can be placed onto the maze in various patterns to create the 6 practice problems and 12 test problems (Figure 2). For the duration of this test, rats were weighed daily with mild food restriction, maintaining no less than 85% of their free-feeding bodyweights; bacon softies (Bio-Serv) were used for motivation and placed in the goal box throughout testing. Testing consisted of three phases: habituation, practice, and test. The habituation phase consisted of 4 days where the rats were first habituated to the apparatus in the absence of any dividing walls and taught to run diagonally to the goal box for a total of eight runs per rat per day. During the practice phase, rats were run through a total of six practice problems for eight trials per day with one new practice problem presented each day. Following, the test phase began where rats were presented with 12 different problems of varying difficulty levels. Rats were presented with one test per day and eight trials per test problem. Each rat had an approximately 2-min inter-trial interval. All test problems were recorded using Ethovision. Errors were recorded by a blind observer and defined as the rat’s head and both front paws passing into a designated error zone. For a diagram of designated error zones per test, see Figure 2. The first trial of each day is considered exploratory in nature as this is the rat’s initial experience with that maze problem. Therefore, errors were counted for trials 2–8 for each test. One rat was excluded from analysis due to failure in training.

### 2.5. Tube Dominance Test

Next, rats underwent a tube dominance task to assess how AIE impacts the presentation of a dominant versus subordinate social status. This task is also relevant to potential human clinical outcomes as low social dominance is evolutionarily linked to poor resource access and enhanced susceptibility to stress-induced depressive phenotypes, termed the “social rank theory” of depression [15,16,17,18]. The tube dominance test is a restrictive dominance test (i.e., forced interaction) [27]. Strategies in this test take into account various processes, including social cognitive processing, aggression, learning and memory, and reward competition. For the duration of this test, rats were mildly food restricted to no less than 85% of their free-feeding bodyweights. Over a series of several days, rats were habituated to the apparatus, which consisted of a Plexiglas tube with a goal box at each end. The center of the tube was divided by two guillotine doors. Rats were trained to approach the guillotine doors upon being placed in the tube, and then proceed to the opposite goal box upon the experimenter lifting the doors whereupon they received a bacon softie. Once all rats immediately approached the doors upon being placed in the apparatus, testing began. Every test day began with a reacquisition trial where the rats were placed in the box and could proceed to the other side where they were awarded a bacon softie. Following reacquisition training, rats were paired against each other in random order. Every AIE rat ran against every control rat each day, with order and start side counterbalanced both across trials and days. Once both rats were in the apparatus and waiting at the center gates, the guillotine doors were lifted and the test began. The test ended when one rat pushed the other out of the tube so that all four paws were outside of the tube. The losing rat was then removed from the apparatus and returned to its home cage. The winning rat was rewarded a bacon softie and then returned to its home cage. The apparatus was wiped clean with Peroxigard between trials. Testing continued for five consecutive days. Videos were recorded with Ethovision and wins per rat per day were recorded by a blind observer.

### 2.6. Novel Object Recognition Test

Lastly, as previous studies have demonstrated a negative impact of AIE on novel object recognition in males in early adulthood [5,11], the current study expanded upon these findings by examining the impact of AIE on novel object recognition in males and females during middle age, 7 months after the end of the AIE paradigm. The open field apparatus consists of an 80 cm × 80 cm black Kydex box with a white floor. All testing was recorded with Ethovision software. The day prior to testing, each rat was habituated to the open field apparatus and allowed to freely explore the environment for 10 min. The chamber was cleaned with Peroxigard between each session to eliminate any potential olfactory cues. The test phase consisted of two days, each with a 5-min testing session. On the first day, rats were placed in the open-field apparatus with two identical objects placed an equal distance from the subject’s starting position. Twenty-four hours later, rats were returned to the apparatus where one of the familiar objects was replaced with a novel one. Familiar and novel objects were counterbalanced across groups and left/right locations. In addition, all objects (i.e., plastic Corning cap, Lego structure) were previously vetted for similar investigative preferences. During both the familiarization and testing phases, the amount of time that the subject was in contact with each object was recorded by a blind observer. Investigation of objects was defined as sniffing, pawing, or having their nose oriented towards the object within 2 cm distance. Accidental interaction with objects through the side of the body or tail were not counted as investigation. Investigation of objects was scored using a timer to count seconds. Object preference was calculated as a percentage of time spent investigating the novel object relative to the familiar object

(1) $$\%\;Preference\;=\;\frac{\left(Time\;Novel\right)}{\left(Time\;Novel\;+\;Time\;Familiar\right)}\times 100$$

### 2.7. Statistical Analyses

All statistical analyses were performed using the statistical software package SPSS. The RAWM was assessed as a 2 × 2 × 20 repeated-measures analysis of variance (RM-ANOVA) with 2 levels of the factor exposure (CON, AIE), 2 levels of the factor of sex (male, female), and 12 levels of the factor trials. Search strategy was assessed over the first five trials using a dummy code of random = 0 or serial = 1 and then analyzed using a RM-ANOVA to see whether exposure and sex interacted over trials to influence search strategy used. The Hebb-Williams maze was assessed as a 2 × 2 × 12 multivariate ANOVA (MANOVA) with 2 levels of the factor exposure (CON, AIE), 2 levels of the factor sex (male, female), and 12 levels of the dependent variable factor test problem. Number of wins for the tube test was assessed as a 2 × 5 RM-ANOVA with two levels of the factor exposure (CON, AIE) and five levels of the factor test day. Males and females were analyzed independently for the tube dominance test and NOR as determined a priori. NOR was assessed using a *t*-test with CON versus AIE. For all tests, α = 0.05. Post-hoc tests were performed when appropriate with a Bonferroni correction. When relevant, results are displayed as the mean ± standard error of the mean (SEM).

## 3. Results

### 3.1. AIE Impairs Spatial Navigation and Alters Search Strategies in the RAWM

**Figure 3:**
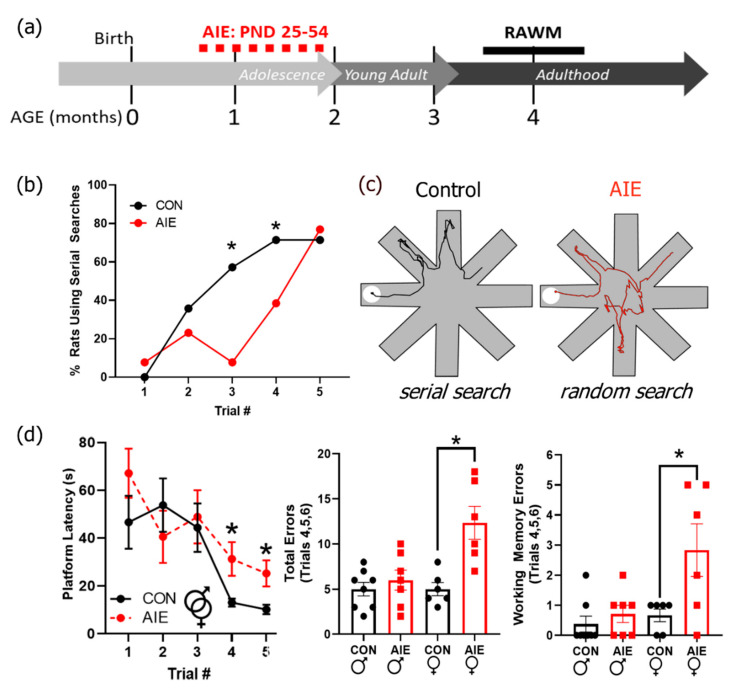
Adolescent intermittent ethanol (AIE) impairs problem-solving flexibility in the radial arm water maze (RAWM). (**a**) Timeline of RAWM paradigm. Rats underwent AIE or control gavage paradigms from PND 25–54. The RAWM was conducted later in adulthood (around 4 months of age). (**b**) Serial versus random search strategies were determined by a blind observer, as described in the methods. Controls increased employment of serial search strategies immediately after the first trial and increasingly employed serial searches on subsequent trials. AIE rats predominantly used random search strategies until trial three (males) and trial four (females), suggesting deficits in problem-solving strategy flexibility. (**c**) On the left is an example of typical run from a control rat using a serial search strategy (trial 5). On the right is an example run from an AIE rat using a random search strategy (trial 5). (**d**) AIE rats took longer to find the platform on the fourth and fifth runs of the first test day, suggesting a mild deficit in spatial learning. This effect is predominantly driven by an increase in the number of total errors made by AIE females. In addition, females tended to make more reference errors, suggesting deficits in working memory may be contributing to these results. Results are expressed as mean ± SEM. * *p* < 0.05.

The RAWM was performed two months after the end of AIE treatment to assess the persistent effects of AIE on spatial navigation and search strategies (Figure 3a). Search strategies typically progress from random to serial searches in spatial navigational tasks prior to retention of platform location where the animal swims directly to the target location. Therefore, we tested whether exposure and sex interacted as a function of trial number to influence search strategy used. Interestingly, there was a significant three-way interaction between AIE treatment, sex, and trial number on search strategy use, *F*(4, 92) = 3.33, *p* = 0.01 (for all RAWM RM-ANOVA results, see Supplementary Table S1). In general, all rats started with a random search strategy and transitioned to a serial search strategy. However, it took longer for females to transition their search strategies from random to serial compared to males, with males beginning that transition by the second trial, *p* = 0.04. AIE treatment delayed the transition from random to serial search strategies in both sexes (Figure 3b,c). Specifically, control males were more likely to use a serial search strategy than AIE males by the third trial, *p* = 0.002, and control females were more likely to use a serial search than AIE females by the fourth trial, *p* = 0.02. These results suggest that AIE impairs behavior by altering flexibility in strategy selection for problem solving.

There was a significant interaction between sex and trial number on latency to find the platform, *F*(19, 437) = 2.54, *p* < 0.001; there was also a trend for a significant interaction between alcohol exposure and trial number on latency to find the platform during acquisition of the RAWM, *F*(19, 437) = 1.58, *p* = 0.057. Follow-up analyses revealed that while AIE-exposed rats took significantly longer to find the platform on the fourth and fifth trials of the first day (Figure 3d), this effect was largely driven by the females. When separated by sex, AIE-treated female but not male rats took significantly longer than control counterparts to find the platform on the fourth and fifth trials of the first day as well as the first trial on the second day, *p*’s = 0.02, 0.01, and 0.03, respectively. When total number of errors was tallied over these three trials, AIE females made significantly more errors than control females, suggesting that the increase in latency in AIE females is largely driven by an increase in incorrect responses, *F*(1, 12) = 13.99, *p* = 0.004. Errors can either be long-term memory errors (i.e., making incorrect arm choice between trials) or working memory errors (i.e., making the same incorrect choice multiple times within a single trial). Female AIE rats made significantly more working memory errors than controls, suggesting that working memory problems in addition to long-term spatial memory problems may be contributing to these differences in both number of errors and overall latency in females, *F*(1, 12) = 5.83, *p* = 0.04. This suggests that AIE mildly impairs acquisition of spatial learning, predominantly in females.

### 3.2. AIE Impairs Working Memory in Hebb-Williams Maze

**Figure 4:**
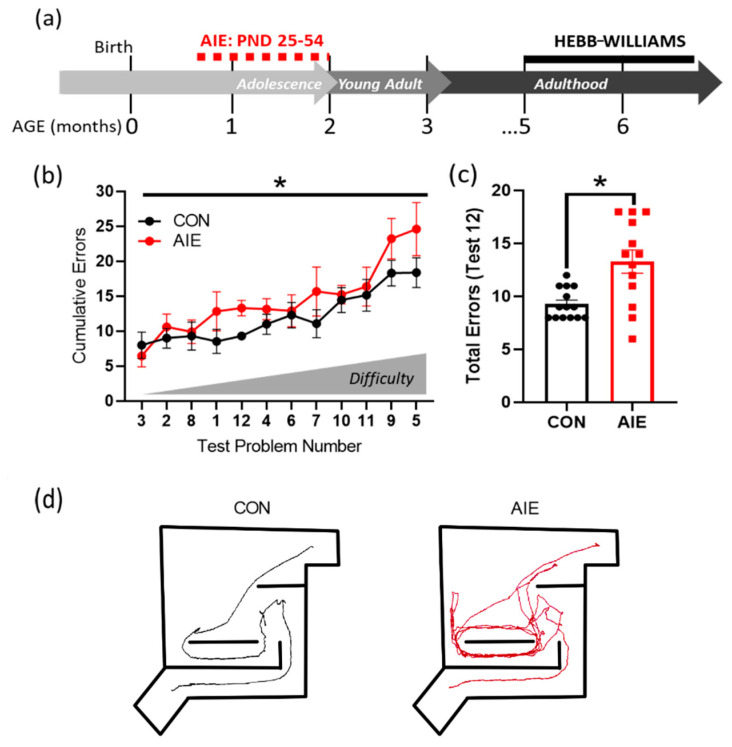
AIE increases errors in the Hebb-Williams maze battery. (**a**) Timeline of Hebb–Williams testing paradigm. Rats underwent AIE or CON gavage paradigms from PND 25–54. The Hebb-Williams maze was conducted later in adulthood (5–6 months of age). Males were tested first, followed by females. (**b**) As shown, tests were ranked by difficulty. In general, AIE increased errors in the Hebb-Williams maze, with greater divergence evident with increasing difficulty. (**c**) An example distribution of the number of errors rats made on test 12. AIE significantly increased the number of cumulative errors across the eight trials. (**d**) Example runs taken from test 12 for control (left) and AIE (right). Controls, on average, did not repeat errors, unlike AIE rats, which perseverated on the same incorrect turns. Results are expressed as mean ± SEM. * *p* < 0.05.

The Hebb-Williams maze was performed more than three months after the end of AIE treatment (Figure 4a); it was used to assess working memory by presenting a series of mazes of varying difficulty levels to solve over the course of 12 consecutive days. Overall, AIE rats made significantly more errors across testing in the Hebb-Williams maze relative to controls, *F*(12, 11) = 2.90, *p* = 0.04 (Figure 4b) (for all Hebb-Williams MANOVA results, see Supplementary Table S2). These effects were the most robust for test 12, where despite only a single potential incorrect turn, AIE rats performed significantly worse than controls, *p* = 0.002 (Figure 4c). In the event of an error, AIE rats repeated the same incorrect response in succession, unlike controls, suggesting a lack of flexibility in correcting incorrect choices within a short timeframe (see Figure 4d for examples). Males and females performed similarly across all tests, indicating that sex did not impact test results, *p* > 0.05. Thus, AIE led to deficits in working memory, as indicated by Hebb-Williams maze performance that lasted long into adulthood.

### 3.3. AIE Increases Submissive Behaviors in Tube Dominance Test

**Figure 5:**
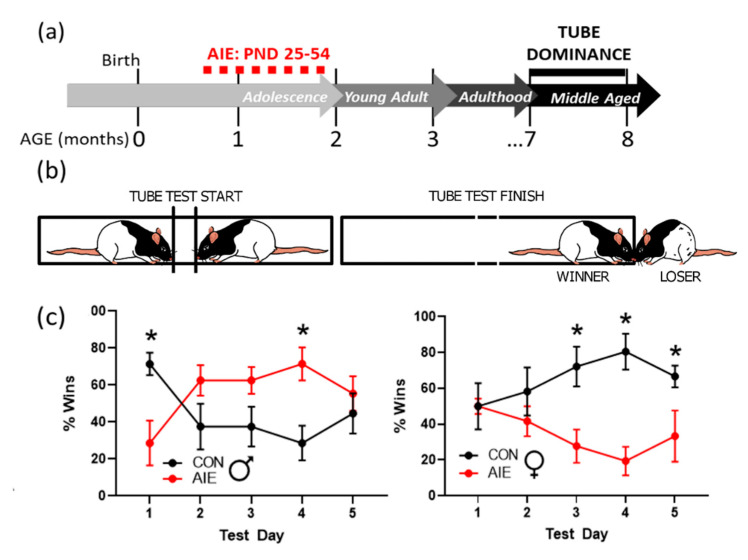
AIE increases submissive responses in the tube dominance test. (**a**) Timeline of tube dominance testing paradigm. Rats underwent AIE or CON gavage paradigms from PND 25–54. The tube dominance test was conducted later in adulthood (7–8 months of age). Males were tested first, followed by females. (**b**) Rats were placed on either side of a clear plexiglass tube and separated by removable gates. The test began when both rats were waiting at the gates, and the gates were removed. The test ended when one rat pushed the other out of the tube. Winning was scored as a binary win-lose. (**c**) Male rats exposed to AIE were more submissive on the initial test day, although this effect was transient where AIE males were more likely to win encounters by the fourth test day. In contrast, female rats exposed to AIE were increasingly more submissive with each test. Results are expressed as mean ± SEM. * *p* < 0.05.

The tube dominance test was performed more than five months after the end of AIE treatment (Figure 5a). This test was used to assess dominance versus submissive behavioral phenotypes following AIE in both male and female rats. As females fail to exhibit aggressive traits in many other social interaction tests (e.g., resident-intruder paradigms), making those tests ill-suited to measure social dominance in females, the tube test is uniquely suited to examine social dominance in both sexes. Results indicate that likelihood of winning at the tube test varies as a function of sex, exposure, and test day, *F*(4, 92) = 11.04, *p* < 0.01. Male AIE rats were more likely to lose to controls on the first day of testing, *p* < 0.05. This effect was reversed on the fourth test day, where male AIE rats were more victorious than their control counterparts, *p* < 0.01. In contrast, AIE-exposed females were progressively more likely to lose as testing continued (Figure 5c). Specifically, female AIE rats were more likely to lose on the third, fourth, and fifth days of testing, *p*’s = 0.01, 0.001, and 0.048, respectively (for an example, see Supplemental Video S1; for all Tube Dominance test RM-ANOVA results, see Supplementary Table S3). Collectively, these results suggest that AIE increases likelihood of losing in both sexes, in a temporally distinct manner.

### 3.4. AIE Impairs NOR in Females

**Figure 6:**
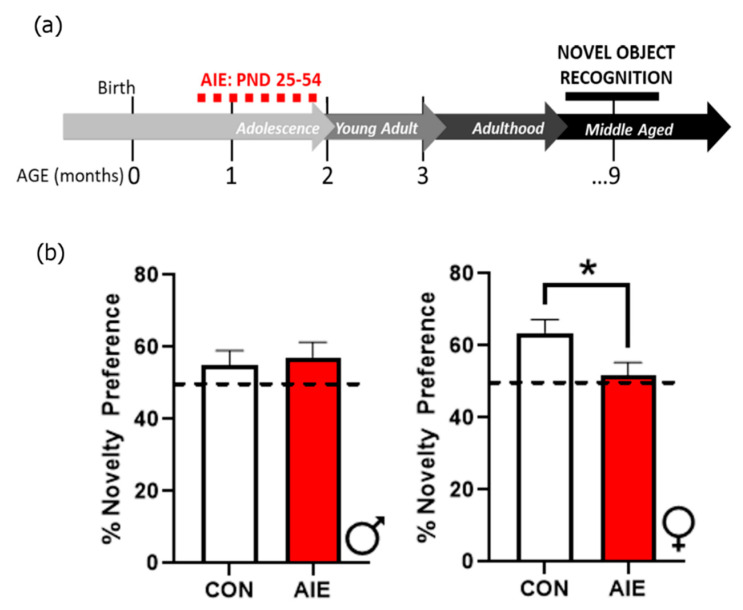
AIE impairs novel object recognition (NOR) in middle age female rats. (**a**) Timeline for NOR. Rats underwent AIE or CON gavage paradigms from PND 25–54. The NOR test was conducted later in adulthood (9 months of age). Males were tested first, followed by females. (**b**) Even in middle age, female rats that were exposed to AIE more than 7 months prior continue to show cognitive deficits, as evidenced by lack of discrimination in the percentage of time spent between novel and familiar objects. Results are expressed as mean ± SEM. * *p* < 0.05.

NOR was performed as a non-spatial test of long-term memory. NOR was also the last behavioral test performed in this battery, where the rats were 9 months old and more than 7 months after the end of AIE (Figure 6a). As previous studies have assessed NOR either immediately or shortly after AIE, this test aimed to examine whether AIE produces cognitive deficits that persist into middle age. Results indicate that there was no effect of AIE on novel object recognition in males, *t*(13) = 0.14, *p* = 0.712. Both AIE and control male rats spent similar amounts of time investigating each object, *t*(13) = 1.80, *p* = 0.20. In contrast, female AIE rats exhibited deficits in time spent with the novel object relative to controls, *t*(10) = 0.68, *p* = 0.04 (Figure 6b). As there were no overarching differences in total investigative time between AIE and control female rats, *t*(10) = 1.48, *p* = 0.25, this indicates that female AIE rats exhibit specific deficits in NOR that are not linked to any overt changes in object interest. These results suggest that females may be particularly sensitive to persistent AIE-induced deficits in NOR, exhibiting deficits even into middle age.

## 4. Discussion

The present study examines the long-term effects of intermittent binge ethanol exposure during adolescence on cognitive-behavioral processes across the lifespan in both male and female rats. Previous studies have not investigated the impact of AIE using the RAWM, Hebb-Williams maze, or tube dominance test. While studies have assessed the impact of AIE on spatial learning in the Barnes maze [6,28], Morris water maze [10], and radial arm maze [29], the RAWM provides unique advantages by combining features of each task, and no study has examined females in these tasks. Similarly, while NOR has been assessed in males and females after alcohol exposure in adolescence, it has not been assessed so far out after the end of the AIE paradigm. Results highlight that (1) adolescent alcohol exposure produces persistent deficits in a battery of working and long-term memory-dependent cognitive tasks as well as dominance behaviors that do not recover despite months of abstinence, further supporting results from previous studies of cognitive deficits using different assessments, and that (2) females appear to be particularly sensitive to AIE long-term cognitive and social hierarchy deficits following adolescent binge drinking.

### 4.1. AIE Impairs Cognitive Functioning and Flexibility

Several studies have indicated that AIE exposure alters cognition and behavior across a variety of domains, including increases in social anxiety in males [12,30,31], increased perseverance during reversal learning in spatial memory tasks [6,9,28], and impaired set-shifting [7]. Conversely, studies have demonstrated that females exhibit increased habitual behavior after AIE [32], and both sexes have evidence of impairments in NOR shortly after the end of AIE exposure [5,20]. An overarching theme from these studies is that cognitive deficits are centered around impairments in learning and behavioral flexibility (i.e., the ability to adapt to changing circumstances or contingencies) [1]. These deficits are most evident in tasks of high complexity [11,33] and manifest through rigidity in cognitive strategies [1,34]. For example, in an operant learning task, AIE-treated males fail to shift to new strategies when new contingencies for reward are put in place (e.g., shifting from a light cue to a lever-pressing based reward system) [7]. Consistent with these findings, we found a failure in strategy shifting after AIE using the RAWM: AIE-treated rats adapted their search strategy from random to serial strategies more slowly than controls. This type of strategy assessment has not yet been assessed in other models of spatial learning, which collectively have suggested no impairments in spatial acquisition. Delayed shifts to serial search strategies instead support a loss of behavioral flexibility during spatial learning tasks that is independent from spatial acquisition deficits, suggesting impairments in proper functioning of executive networks.

As working memory is a subcomponent of executive functioning, these studies support previous findings of AIE-induced long-lasting deficits in this cognitive domain. We expand upon these previous studies with our finding that AIE produces significant impairments in working memory function across multiple behavioral tasks. In the RAWM, female AIE rats made significantly more working memory errors than control counterparts. In addition, both males and females made significantly more errors across trials in the Hebb-Williams maze, which directly assesses working memory as a function of task difficulty. Amazingly, 14 doses of intermittent ethanol exposure across adolescence produced problem solving deficits in the Hebb-Williams maze similar in magnitude to deficits evidenced by 150 days of liquid diet across adulthood [35], highlighting the vulnerability of the adolescent brain to persistent cognitive deficits. Consistent with findings from previous AIE studies, AIE-treated rats performed similarly on easy maze problems but were impaired as maze difficulty increased, further indicating that task difficulty is an important component of assessing cognitive deficits after AIE. Like in the RAWM task, in the Hebb-Williams maze, AIE rats made repetitive errors within a single trial. This was particularly evident in the twelfth maze, where there was only one potential incorrect path. Repeating errors in a short timeframe suggests that AIE may be impairing the brain’s ability to update information appropriately in working memory, further emphasizing deficits in executive functioning networks.

Interestingly, while both male and female AIE-treated rats exhibited deficits in spatial navigation and search strategies in the RAWM and working memory function as assessed via the Hebb-Williams maze, female AIE rats also exhibited deficits in long-term memory performance in a novel object recognition task more than seven months after the end of alcohol exposure. Of note, while AIE did not impair NOR in males, this is largely due to poor object retention in both AIE and control groups at this age. Decreased discrimination between novel and familiar objects in NOR tasks have been documented after AIE in males at PND 165 [5], and females exhibit similar novel object discrimination failures after adolescent two-bottle choice alcohol exposure, which lasts at least until early adulthood (PND 60) [20]. Until now, no study has determined whether AIE impacts novel object discrimination in NOR in both sexes during middle age (>7 months). The persistence of these deficits in females suggests that adolescent exposure to alcohol permanently alters neural circuits driving novel object recognition, and that these deficits are not reversible in the event of abstinence. In addition, the persistence of NOR deficits after AIE in females suggests that adolescent alcohol intake permanently changes the developmental trajectory of the brain and, consequentially, cognitive processes. This could indicate that AIE is a risk factor for early cognitive decline, later alcohol use disorder, or the development of other emotional disorders (e.g., depression).

The RAWM, the Hebb-Williams maze, and the NOR tasks all rely heavily on a combination of proper functioning between the prefrontal cortex and the hippocampus. We have previously demonstrated that AIE males exhibit increased neuroinflammation in both brain regions relative to controls [5,9]. For example, our lab has previously identified that toll-like receptor 3 and 4 as well as high-mobility group box 1 (HMGB1) expression are upregulated in the medial PFC after AIE in males, indicating increases in neuroinflammation that may underlie some of the deficits in executive functioning [9]. Similarly, AIE increases HMGB1 and TNF-α mRNA in the hippocampus in males, relative to control counterparts, potentially contributing to long-term memory deficits after AIE [5]. This could suggest that a persistent induction of neuroinflammation is a critical underlying factor driving long-term cognitive deficits after AIE, and that interventions targeting neuroinflammation may be critical to reverse these deficits. In support of this hypothesis, increased perseveration during reversal learning in the Morris water maze can be prevented in males by using anti-inflammatory drugs such as indomethacin [21] or reversed through exercise [10,21]. However, it is important to note that neuroinflammation in the PFC or hippocampus of AIE-exposed females has not yet been confirmed and remains an important future direction for the field.

### 4.2. AIE Increases Submissive Phenotypes, Indicating Potential Emotional Dysregulation and Stress Susceptibility

In addition to impairing working and long-term memory tasks, studies have indicated that AIE may also disrupt social behaviors [12,36]. One of the most fundamental behaviors in social species is the establishment of an organized social hierarchy [37]. Social hierarchy establishment is highly conserved across social species, and in many cases low rankings in social hierarchy result in poor health outcomes through increased stress susceptibility as well as poor resource access (e.g., food, water, shelter, and mates), making social status a driving factor of natural selection [15]. While social dominance and submission in hierarchies may appear domain-specific from other cognitive modalities, the establishment of social hierarchies is highly dependent on a variety of cognitive functions, and some studies have indicated that environmental factors that shift rats to a subordinate phenotype also produce a host of other cognitive deficits [38]. This suggests that while dominant-submissive behaviors in the tube dominance test and cognitive behaviors in NOR, RAWM, and the Hebb-Williams maze may appear domain-specific, there exists a critical underlying link between them. As such, the ability of AIE to alter dominance phenotypes has important potential implications for cognitive function as well as stress susceptibility and the development of other co-morbid disorders such as depression [19]. In support of this hypothesis, alcohol exposure during adolescence increases depressive-like phenotypes in males, as evidenced by increased immobility in the forced swim test [39] in addition to increasing stress-induced drinking behavior in adulthood in both male and female rats [40,41]. These results are unique from social investigation and social preference tasks, where deficits are primarily evidenced in males [12,30,31]. Divergence between social investigation and dominance emphasizes the importance of distinguishing a forced confrontation stressor as a contributing factor in behavioral outcomes.

In the current study, while males exhibited a transient submissive phenotype in the tube dominance test relative to control males, AIE females exhibited an increasingly submissive phenotype compared to control females across testing, suggesting that females may be particularly sensitive to alcohol-induced effects on the social hierarchy in conflict situations when evaluated against same-sex partners. Like many of the cognitive tests on working memory, social dominance and submission are also regulated by proper prefrontal cortical function [42], suggesting that investigation of the effects of AIE on PFC activity is a critical future direction, particularly in females. In support of this, AIE decreases connectivity between the PFC and limbic structures in rodent studies using function magnetic resonance imaging [43]. Similarly, human studies indicate that the PFC is impacted by adolescent alcohol intake in a sex-specific manner since female teenagers with alcohol use disorder have smaller PFC volumes whereas males with alcohol use disorder have larger PFC volumes relative to age-matched healthy controls [44]. In contrast, the transition from an originally submissive phenotype in AIE-treated male rats to an increase in dominance behaviors is more complicated to interpret. One could be tempted to explain the paradoxical effects of AIE on increasing dominant phenotypes over time as reflective of resilience against a sensitivity to the induction of a depressive phenotype. Indeed, other studies have similarly indicated that alcohol can produce paradoxical effects in the brain with regards to depression, such as its ability to normalize the brain’s lipid imbalance in transgenic models of depression [45]. However, an important caveat of this interpretation is that increases in dominant phenotypes can also be reflective of general aberrations in appropriate social interactions as well an imbalance within neuromodulatory systems, including prostaglandin [46] and acetylcholine [47,48]. Indeed, several studies that have examined social interaction behaviors after AIE demonstrated that males but not females exhibit deficits in non-confrontational social interactions [12,30], suggesting that the paradoxical effects of the tube dominance in males may be reflective of more complex effects on social behavior. Future studies should also expand on these findings to examine whether interventions that reverse cognitive deficits after AIE also reverse AIE-induced changes in social hierarchy behaviors.

## 5. Conclusions

Collectively, these results indicate that adolescent alcohol exposure produces permanent but modest deficits on cognitive function across a battery of behavioral paradigms, highlighting the need for adolescent alcohol intake in humans to be re-evaluated as a contributing factor for the development of later comorbidities, including substance abuse disorders, mood disorders, and early cognitive decline. In particular, deficits in behavioral flexibility are a core component of both alcohol use disorder and depression, suggesting that adolescent alcohol exposure may be a risk factor for later development of other psychological disorders. As men generally have higher rates of alcohol use disorder whereas females generally have higher rates of depression, adolescent alcohol exposure may also contribute to the pathophysiology underlying these sex differences. Lastly, as increased neuroinflammation is a common feature across these potential comorbidities, AIE-induced neuroinflammation may be a core underlying feature of the developmental physiopathology in some populations. This suggests that examining whether reversing AIE-induced neuroinflammation in males and females through interventions like exercise also reverses cognitive deficits may be an important future direction for the field, and that binge drinking during adolescence may produce long-lasting neurological effects beyond those that clinical studies typically examine.

## Supplementary Materials

The following are available online at https://www.mdpi.com/2076-3425/10/11/785/s1, Table S1: Radial Arm Water Maze ANOVA Results; Table S2: Hebb-Williams Maze ANOVA Results; Table S3: Tube Dominance Test ANOVA Results; Video S1: AIE increases submissive behaviors in females.
