# Supplementary material for: Adolescent Alcohol Exposure Produces Protracted Cognitive-Behavioral Impairments in Adult Male and Female Rats
Source: Brain Sci. 2020 Oct 28;10(11):785. doi: 10.3390/brainsci10110785 (PMC7692738; doi:10.3390/brainsci10110785)
Supplement: Supplementary file 1 [file brainsci-10-00785-s001.zip › SupplementalTables1_2_3.docx]

| *Supplemental Table 1: Radial Arm Water Maze ANOVA Results* | | | | | | | |
| --- | --- | --- | --- | --- | --- | --- | --- |
|  | **Search Strategy** | | |  | **Latency** | | |
| *Factors* | *df* | *F* ratio | *p* value |  | *df* | *F* ratio | *p* value |
| Sex × Exposure × Trial | 4, 92 | 3.33 | 0.01 |  | 19, 437 | 0.79 | 0.72 |
| Sex × Trial | 4, 92 | 2.05 | 0.09 |  | 19, 437 | 2.54 | <0.001 |
| Exposure × Trial | 4, 92 | 1.48 | 0.21 |  | 19, 437 | 1.58 | 0.057 |
| Trial | 4, 92 | 10.78 | <0.001 |  | 19, 437 | 16.60 | <0.001 |
| Sex × Exposure | 1, 23 | 0.55 | 0.47 |  | 1, 23 | 1.18 | 0.29 |
| Sex | 1, 23 | 0.71 | 0.41 |  | 1, 23 | 15.09 | 0.001 |
| Exposure | 1, 23 | 2.09 | 0.16 |  | 1, 23 | 1.89 | 0.18 |

| *Supplemental Table 2: Hebb-Williams Maze ANOVA Results* | | | |
| --- | --- | --- | --- |
|  | **Errors** | | |
| *Factors* | *df* | *F* ratio | *p* value |
| Sex × Exposure | 12, 11 | 0.79 | 0.65 |
| Exposure | 12, 11 | 2.90 | 0.04 |
| Sex | 12, 11 | 1.10 | 0.46 |

| *Supplemental Table 3: Tube Dominance Test ANOVA Results* | | | | | | |  |
| --- | --- | --- | --- | --- | --- | --- | --- |
|  | **Errors** | | | | | |  |
| *Factors* | *df* | | *F* ratio | | *p* value | |  |
| Sex × Exposure × Test Day | | 4, 92 | | 11.04 | | <0.001 | |
| Sex × Test Day | 4, 92 | | 0.00 | | 1.00 | |  |
| Exposure × Test Day | 4, 92 | | 1.25 | | 0.30 | |  |
| Test Day | 4, 92 | | 0.00 | | 1.00 | |  |
| Sex × Exposure | 1, 23 | | 9.53 | | 0.01 | |  |
| Sex | 1, 23 | | 0.00 | | 1.00 | |  |
| Exposure | 1, 23 | | 1.83 | | 0.19 | |  |
